# Corneal Nerve Parameter Reference Values for Chinese Adults Assessed by Corneal Confocal Microscopy

**DOI:** 10.1155/2022/4913031

**Published:** 2022-02-27

**Authors:** Juan Cao, Jingru Qu, Bekzod Odilov, Bin Lu, Yuanpin Zhang, Lili Li, Yuan Zhang, Qian Xiong, Yu Hong, Jianqiao Li, Yunfeng Shen, Xinguo Hou

**Affiliations:** ^1^Department of Endocrinology, Qilu Hospital, Cheeloo College of Medicine, Shandong University, Jinan, Shandong 250012, China; ^2^Department of Health Management Center, Qilu Hospital of Shandong University, 107 Wenhua W Road, Jinan, Shandong 250012, China; ^3^Institute of Endocrine and Metabolic Diseases of Shandong University, 107 Wenhua W Road, Jinan, Shandong 250012, China; ^4^Key Laboratory of Endocrine and Metabolic Diseases, Shandong Province Medicine & Health, 107 Wenhua W Road, Jinan, Shandong 250012, China; ^5^Jinan Clinical Research Center for Endocrine and Metabolic Diseases, Shandong Province Medicine & Health, 107 Wenhua W Road, Jinan, Shandong 250012, China; ^6^Department of Endocrinology and Metabolism, Huashan Hospital, Fudan University, 12 Wulumuqi Middle Road, Shanghai 200040, China; ^7^Department of Ultrasound, Qilu Hospital of Shandong University, 758 Hefei Road, Qingdao, Shandong 266000, China; ^8^Clinical Epidemiology Unit, Qilu Hospital of Shandong University, 107 Wenhua W Road, Jinan, Shandong 250012, China; ^9^Clinical Research Center of Shandong University, 107 Wenhua W Road, Jinan, Shandong 250012, China; ^10^Department of Endocrinology and Metabolism, Gonghui Hospital, 315 Shimen No. 1 Road, Shanghai 200040, China; ^11^Department of Ophthalmology, Qilu Hospital of Shandong University, 107 Wenhua W Road, Jinan, Shandong 250012, China; ^12^Department of Endocrinology and Metabolism, Institute for the Study of Endocrinology and Metabolism in Jiangxi Province, The Second Affiliated Hospital of Nanchang University, 1 Minde Road, Nanchang, Jiangxi 330006, China

## Abstract

**Background:**

Confocal corneal microscopy is an excellent new noninvasive tool for assessing diabetic peripheral neuropathy. We aimed to investigate the clinical variables associated with corneal nerve parameters and establish reference values for clinical use in healthy Chinese adults.

**Methods:**

The study enlisted 257 healthy volunteers (137 females and 120 males) from two clinical academic centers in China. Two experts captured and selected images of the central corneal subbasal nerve plexus at each center using the same corneal confocal microscopy instrument according to a commonly adopted protocol. Corneal nerve fiber density (CNFD), corneal nerve branch density (CNBD), and corneal nerve fiber length (CNFL) were measured using fully automated software (ACCMetrics). The correlation between clinical indicators and confocal corneal microscopy measures was determined using partial correlation. Quantile regression was used to calculate reference values and estimate the effects of clinical factors on the normative values of confocal corneal microscopy measures.

**Results:**

Females had significantly higher CNFD, CNBD, and CNFL than males. There was no correlation between age, glycated hemoglobin (HbA1c), height, weight, body mass index (BMI), and any corneal nerve fiber parameter in both sexes. In either sex, age, weight, height, BMI, and HbA1c did not affect the 0.05^th^ quantile values of any corneal nerve parameter.

**Conclusions:**

This study establishes sex-adjusted reference values for corneal confocal microscopy measures in Chinese adults and provides a reference for clinical practice and research with this technique.

## 1. Introduction

Diabetic peripheral neuropathy (DPN), for which there is currently no effective therapy, affects nearly 50% of people with type 2 diabetes mellitus [1]. However, the most common assessment methods for DPN have substantial limitations, such as poor objectivity [2], insensitivity to early diagnosis [3], or invasiveness [4]. The application of corneal confocal microscopy (CCM) may provide a fast, reliable, and noninvasive alternative method for detecting and diagnosing peripheral neuropathies, especially DPN. In recent years, many studies have demonstrated that CCM has good sensitivity and specificity in diagnosing DPN [5–8]. It has been recommended as a surrogate endpoint for DPN assessment [9].

As an ideal tool for the noninvasive assessment of DPN, establishing normative reference values for CCM is urgently needed. Standardizing protocols for capturing, sampling, and analyzing corneal nerve images is essential. Many studies have investigated these criteria and developed a commonly used protocol [10, 11]. In a recent multicenter study, Tavakoli et al. established the age-adjusted normative values in Western populations using a common method to obtain images and manual image analysis software [12]. In previous studies, reports on the association between age and corneal nerve morphology in healthy individuals are conflicting [13–15]. In addition, a standardized, automated image analysis program (ACCMetrics) has been widely used [16, 17]. It allows for the objective and rapid calculation of strictly defined corneal nerve parameters, avoiding inter- and intraobserver differences in manual analysis and allowing for study comparisons [18]. Therefore, we evaluated corneal nerve parameters in Chinese adults using ACCMetrics in multiple research centers to investigate the effect of clinical factors on CCM measures and establish reference values for CCM parameters in Chinese adults.

## 2. Materials and Methods

### 2.1. Participants

According to the Clinical and Laboratory Standards Institute Guidelines, the minimum sample size to determine the 5^th^ percentile reference interval is 120 if the study calculated the reference interval using a nonparametric method [19, 20]. The current study required a minimum of 120 samples per gender group because the reference intervals were stratified by sex. We included 257 healthy volunteers (120 males and 137 females) from two independent clinical research centers (Jinan, *n* = 163; Shanghai, *n* = 94). All participants were Chinese, in good health, and ranged from 18 to 85 years.

They were selected from residents managed by the hospital health management center and the community health service center, who received a comprehensive annual health check-up and health education. A physician initially screened the volunteers through face-to-face interviews and physical examinations in each center. The questionnaire included demographic information; history of present illness; recent symptoms of fatigue, anxiety, palpitations, fever, and other subhealth status; past medical, family, and drug-allergy history; alcohol abuse; regular activity; diet; and sleep. Physical examination mainly includes height, weight, blood pressure, cardiopulmonary auscultation, abdominal palpation, and thyroid palpation. Blood samples for glycated hemoglobin (HbA1c), fasting plasma glucose (FPG) and other tests required for an annual check-up (including complete blood count, liver function tests, and kidney function tests, etc.) were sent to the hospital clinical laboratory for testing.

Participants with diabetes, impaired fasting glycemia, or prediabetes were excluded based on HbA1c and FPG [21]. Volunteers with acute disease, history of chronic gastrointestinal disease, hypertension, cerebrovascular disease, malignant tumor, autoimmune disease (systemic lupus erythematosus, systemic sclerosis, Crohn's disease, etc.), vitamin B12 or folate deficiency, hypothyroidism, hepatic or renal dysfunction, cervical or lumbar spine disease, and central neurodegenerative diseases (including Parkinson's disease, multiple sclerosis, Alzheimer's disease, Huntington's disease, and dementia) were excluded. Those with a family history of genetic disorders (including but not limited to hereditary neuropathy), history of ocular trauma, diseases, corneal disorders, surgery, contact lens wearing, alcohol abuse, and toxic or chemotherapeutic drug exposure were also excluded. Pregnant or breastfeeding women and vegetarians were excluded from the study.

### 2.2. Demographic, Medical, and Laboratory Data

All participants' sex, age, height, weight, FPG, and HbA1c were collected. All volunteers who passed the initial screening underwent neurological scoring systems (neuropathy symptom score (NSS) [22], neuropathy disability score (NDS) [22], Michigan neuropathy screening instrument (MNSI) [23], Toronto clinical scoring system (TCSS) [24]), and detailed peripheral nervous system examinations by an experienced neurophysiologist at each center. Peripheral nervous system examination includes a vibration test by 128 Hz tuning fork, temperature sensation, 10 g Semmes-Weinstein monofilament examination, superficial pain sensation, and ankle jerk reflex. NC-STAT DPNCheck (NeuroMetrix, Waltham, MA) was used to detect sural nerve conduction in both lower extremities, and two parameters were recorded: sural nerve conduction velocity (SNCV) and sural nerve conduction amplitude (SNCA). A score of 0 in all four scoring systems was considered normal; the normal reference range for nerve conduction examination is SNCV ≥ 41 m/s and SNCA ≥ 5 *μ*V. Individuals with abnormal findings were excluded. This study was reviewed by the Ethics Committee of Qilu Hospital of Shandong University and adhered to the principles of the Declaration of Helsinki. All participants gave informed consent before their participation.

### 2.3. *In Vitro* Confocal Corneal Microscopy and Analysis of Nerve Fiber Images

All participants underwent examination with the Rostock Corneal Module of the Heidelberg Retinal Tomograph III (Heidelberg Engineering, Heidelberg, Germany) by two experts according to a previously established protocol in each center [25]. The participant's right eye was anesthetized with a drop of 0.4% oxybuprocaine hydrochloride. A drop of hydroxypropyl methylcellulose was placed in the center of the objective lens. On the objective lens, a sterile corneal cap was placed. The lens was slowly moved until it touched the cornea, and then, the focal plane adjustment ring was turned to obtain images of various depths and multiple points. Three to six nonoverlapping, high-quality, and high-contrast images of the central cornea subbasal nerve plexus (SNP) were selected for analysis.

**Figure 1:**
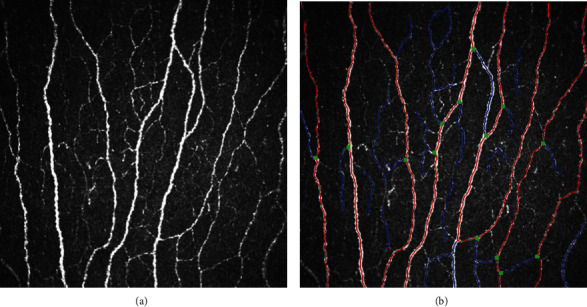
A representative *in vitro* corneal confocal microscopy image and the corresponding analyzed image in a healthy participant. (a) A representative *in vitro* corneal confocal microscopy image of a healthy participant's central corneal subbasal nerve plexus. (b) The corresponding nerve fiber image analyzed by the automated software (ACCMetrics, University of Manchester, Manchester, UK). Red lines indicate the main corneal nerve fibers, corneal nerve branches are represented in blue, and branch points are shown in green dots.

Fully automated analysis software (ACCMetrics) was used to quantify corneal nerve morphology [18]. Three commonly used parameters were calculated: corneal nerve fiber length (CNFL; total nerve fiber length/mm^2^), corneal nerve fiber density (CNFD; the number of main nerve fibers/mm^2^), and corneal nerve branch density (CNBD; the total number of main nerve branches/mm^2^). A representative *in vitro* CCM image and the corresponding analyzed image are shown in Figure 1.

### 2.4. Statistical Analyses

Statistical tests were performed using Stata MP16.0 (StataCorp, College Station, TX). The Kolmogorov-Smirnov test and histograms were used to verify the normality of the dataset. Potential correlations were assessed using the Spearman rank correlation analysis or Pearson correlation coefficient. Variables related to CCM parameters were evaluated by partial correlation analysis and multiple linear regression. Comparisons between sexes were assessed using the Mann–Whitney *U* test or independent sample *t* test. The Kruskal-Wallis rank sum test or one-way analysis of variance was used to compare different age groups. The effects of clinical variables on corneal nerve parameter quantiles and normative values were calculated by quantile regression. Quantile regression does not depend on distributional assumptions, which is significantly robust to outliers and skewness [26]. Statistical significance was established at *P* < 0.05.

## 3. Results

There were 257 healthy volunteers included in this study, stratified by age and sex (at least 15 individuals per decade, 120 males (46.7%) and 137 females (53.3%)). The ages of the individuals ranged from 18 to 85 (mean age, 52.8 ± 16.2) years. Clinical and demographic data for each age group are presented in Table 1.

Correlation analysis indicated that CNFD (*r* = 0.17, *P* = 0.007), CNFL (*r* = 0.16, *P* = 0.011), and CNBD (*r* = 0.12, *P* = 0.048) were significantly correlated with sex and CNFD (*r* = −0.13, *P* = 0.044) was significantly correlated with height. Sex and height were entered into the stepwise multiple linear regression model, and it was found that only sex entered the model and showed a significant correlation with CNFD. Partial correlation analysis (adjusted for sex) between CCM parameters and all clinical variables showed no statistical significance (Table 2). CNFD, CNBD, and CNFL were significantly higher in females than in males (*P* = 0.007, 0.049, and 0.006, respectively). Descriptive statistics for the distribution of corneal nerve fiber parameters in male and female participants are presented in Table 3.

In either the male or female groups, there was no correlation between height, weight, BMI, FPG, or HbA1c and any CCM parameter. Neither BMI, weight, height, FPG, nor HbA1c affected the normative reference values (0.05^th^ quantiles of CNFD, CNBD, and CNFL) in either sex.

**Figure 2:**
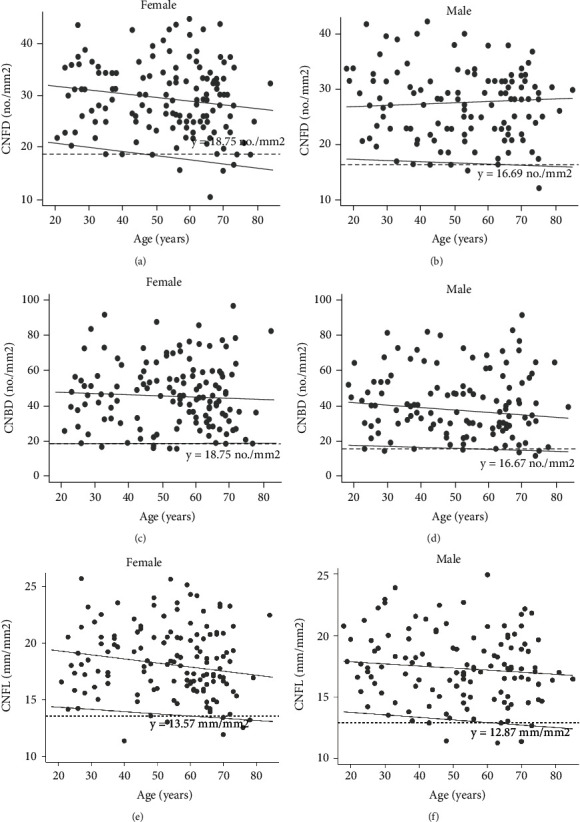
Scatterplot showing corneal nerve parameter values in 257 healthy volunteers stratified by sex (120 males, 137 females). (a) CNFD for female. (b) CNFD for male. (c) CNBD for female. (d) CNBD for male. (e) CNFL for female. (f) CNFL for male. Black continuous lines describe the 5^th^ and 50^th^ quantile regression lines. Red dotted lines represent reference values for corneal nerve parameters we recommend.

Age was not associated with CNFD (*r* = −0.088, *P* = 0.159), CNBD (*r* = −0.033, *P* = 0.602), or CNFL (*r* = −0.114, *P* = 0.069). There were no significant differences in CCM measures among age groups in either sex. Age had no effect on the 0.05^th^ or 0.5^th^ quantile values for any CCM measure in either sex in the quantile regression analysis.  Figure 2 depicts the scatterplots of each CCM parameter with age for male and female participants. Sex-adjusted corneal nerve parameter reference values and their 95% confidence intervals for adults are presented in Table 4.

## 4. Discussion

DPN is a common chronic complication of diabetes. Up to 50% of people with DPN may be asymptomatic, putting them at risk for foot injuries if they are not detected early and given preventive care [27]. The evidence supporting the use of CCM in screening DPN is robust [9]. This study investigated the corneal nerve reference values for clinical use in Chinese adults, following a commonly adopted protocol and using a fast, automated analysis program.

This study found neither BMI, height, weight, nor HbA1c affected corneal nerve parameters or 5^th^ percentile reference values in either sex. The results of BMI, height, and weight were consistent with those of previous studies [12, 15]. We found no significant correlation between corneal nerve parameters and HbA1c, which is consistent with Tavakoli et al. [12]. However, Wu et al. [15] showed that HbA1c was the only variable associated with CNFL in the multiple regression model. These differences may be related to different image selection and analysis methods or may be partly because none of these studies conducted oral glucose tolerance tests to exclude potential prediabetics.

The effect of age on CCM parameters in healthy people is still debated. Several studies have found that as people get older, their corneal nerve density (as measured by CCM) decreases [12, 28], while others have not found this correlation [14, 29]. It should be noted that previous studies did not unify corneal nerve parameters, but the concept of corneal nerve density in these studies was consistent with the definition of CNFL [28–30]. Noticeably, the use of different definitions of corneal nerve parameters and different CCM types and image selection methods may have influenced previous findings. However, the influence of age also showed conflicting results in an *in vitro* study of human corneas. He et al. [31] discovered that central epithelial nerve density decreased with age after generating a three-dimensional map using 22 fresh human corneas. In contrast, Marfurt et al. [32] studied 16 donor corneas aged 19–78 years and discovered no significant correlation between donor age and SNP density.

It is crucial to understand whether corneal nerve parameters are independent of age, as this determines whether age stratification is required when establishing CCM index reference values. A cross-sectional study of 108 healthy corneas showed that the number, density, and beading number of the SNP were not significantly associated with age [14]. Wu et al. [15] found that CNFD and CNBD were not significantly associated with age and CNFL was only independently associated with HbA1c, not with age. Tavakoli et al. [12] indicated an independent age-related reduction in CNFD and CNFL in a recent international multicenter collaborative study. However, the recommended 5^th^ percentile cutoff points of CNFL and CNFD for clinical use in that study only showed a correlation in the youngest group (9-16 years), with no evident correlation in older people (>26 years). That may suggest that the normative reference values of CNFL and CNFD are more strongly correlated in adolescents than adults. Most participants in our study were over 25 years of age, and no minors were included, which might affect the significance of the correlation between age and CCM measures. Large-high-quality longitudinal studies are needed to demonstrate the influence of age on corneal nerve morphology.

An intriguing finding of this study is that the corneal nerve parameter values were lower in males than females, which is similar to the findings of studies on intraepidermal nerve fiber density (IENFD) [33, 34]. In contrast, earlier studies have not reported a sex-dependent difference in corneal nerve metrics [12, 13, 35]. Similar to IENFD, these sex differences may be related to hormonal differences. Progesterone has been shown to promote axonal growth and myelin formation [36]. Kovacic et al. [37] found that peripheral nerves in female rats sprouted and regenerated more quickly after injury. A recent experimental study showed that female mice have a faster rate of corneal nerve regeneration than male mice because female corneas secrete more neurotrophic factors that modulate the expression of related genes [38]. Additionally, *β*-estradiol stimulated the secretion of neurotrophins in the tears of injured mice, and topical treatment with *β*-estradiol promoted the mice corneal nerve regeneration process and resulted in increased subbasal nerve density. Furthermore, a previous study has demonstrated a negative correlation between chronic smoking and corneal SNP fiber numbers [39]. In China, men are known to smoke more cigarettes than women, accounting for the neuropathic effect. The lack of detailed data on smoking limits our study that merits further investigation.

There are other limitations to our study, such as the lack of young people and the fact that these volunteers were not representative of the actual population. Furthermore, we did not perform oral glucose tolerance tests on all participants to assess their metabolic status. ANA, IgGs, B12, and folate levels were not tested; individuals with autoimmune diseases were only excluded through a detailed questionnaire and physical examination. In addition, because vitamin B12 and folate deficiencies can cause megaloblastic anemia, volunteers with abnormal complete blood count were excluded; vegetarians, dieters, and volunteers with chronic gastrointestinal disorders were also excluded. These may indirectly eliminate vitamin B12 and folate deficiency.

## 5. Conclusions

In conclusion, healthy people were strictly screened through questionnaire survey and systematic physical examination. This study represents the initial cohort of healthy Chinese adults with CCM data and provides age-independent normative reference values for corneal nerve parameters for this population. This study provides a basis for the widespread application of CCM in related clinical diagnosis and research.

## Figures and Tables

**Table 1:** Demographic characteristics of the participants.

	Age groups (years)						
	18-29	30-39	40-49	50-59	60-69	≥70	Total
Number (male/female)	18/15	17/16	16/19	20/25	26/43	23/19	120/137
Height (m)	1.70 ± 0.09	1.68 ± 0.08	1.67 ± 0.09	1.66 ± 0.07	1.65 ± 0.07	1.65 ± 0.08	1.66 ± 0.08
Weight (kg)	64.8 ± 15.9	64.4 ± 12.1	67.0 ± 10.2	66.2 ± 9.4	65.4 ± 10.6	62.5 ± 10.4	65.1 ± 11.3
BMI (kg/m^2^)	22.2 ± 3.8	22.8 ± 3.2	24.1 ± 2.4	24.0 ± 2.4	24.0 ± 3.0	22.8 ± 2.5	23.4 ± 3.0
FPG (mmol/L)	4.9 ± 0.4	5.0 ± 0.4	5.1 ± 0.4	5.2 ± 0.3	5.2 ± 0.4	5.1 ± 0.4	5.1 ± 0.4
HbA1c (%)	5.1 ± 0.3	5.2 ± 0.4	5.2 ± 0.3	5.3 ± 0.3	5.5 ± 0.3	5.5 ± 0.3	5.3 ± 0.3
Right SNCV (m/s)	56.27 ± 4.22	56.73 ± 4.84	54.97 ± 4.57	53.40 ± 3.66	57.78 ± 5.05	57.21 ± 5.82	56.21 ± 4.99
Right SNAP (*μ*V)	18.88 ± 3.73	17.73 ± 3.43	18.54 ± 4.08	16.22 ± 4.89	14.97 ± 6.08	13.48 ± 4.65	16.29 ± 5.14
Left SNCV (m/s)	56.06 ± 14.43	56.67 ± 4.79	54.17 ± 5.01	53.24 ± 3.89	58.04 ± 5.31	56.98 ± 6.34	56.07 ± 5.26
Left SNAP (*μ*V)	18.97 ± 3.69	17.88 ± 3.54	18.51 ± 3.74	17.44 ± 5.47	14.65 ± 5.99	14.05 ± 5.66	16.54 ± 5.35

Data are presented as mean ± standard deviation. BMI: body mass index; FPG: fasting plasma glucose; HbA1c: glycated hemoglobin; SNCV: sural nerve conduction velocity; SNAP: sural nerve amplitude.

**Table 2:** Partial correlation between CCM measures and clinical variables (adjusted for sex).

	CNFD		CNBD		CNFL	
	*r*	*P* value	*r*	*P* value	*r*	*P* value
Age	-0.108	0.084	-0.019	0.759	-0.117	0.062
HbA1c	0.008	0.894	-0.045	0.474	-0.031	0.616
FPG	-0.002	0.979	-0.016	0.799	-0.011	0.855
BMI	0.046	0.464	0.082	0.194	0.017	0.790
Height	-0.023	0.717	0.035	0.578	0.030	0.639
Weight	0.026	0.679	0.089	0.157	0.026	0.677

*r*: partial correlation coefficient; CNFD: corneal nerve fiber density; CNBD: corneal nerve branch density; CNFL: corneal nerve fiber length; HbA1c: glycated hemoglobin; FPG: fasting plasma glucose; BMI: body mass index.

**Table 3:** Descriptive statistics for the distribution of corneal nerve fiber parameters in male and female participants.

	Sex	5^th^	10^th^	25^th^	50^th^	75^th^	90^th^	95^th^	Mean ± SD	*P* value
CNFD	Male	16.69	18.75	21.87	27.60	31.25	34.37	38.49	26.97 ± 6.22	0.007
	Female	18.75	20.21	25.00	29.16	34.37	38.54	42.71	29.30 ± 6.94	
CNBD	Male	16.67	18.75	30.05	39.84	54.55	69.68	77.91	42.41 ± 18.49	0.049
	Female	18.75	22.71	32.29	45.83	57.81	73.12	78.54	46.54 ± 18.31	
CNFL	Male	12.87	13.60	15.32	17.27	19.51	21.33	22.46	17.40 ± 2.86	0.006
	Female	13.57	14.59	16.29	18.16	20.60	23.03	23.62	18.43 ± 3.08	

Data are presented as centiles and mean ± SD (standard deviation) for corneal nerve fiber parameters. *P* value refers to the male vs. female group.

**Table 4:** Corneal nerve parameter reference values.

	5^th^ centile CNFD (no./mm^2^)	5^th^ centile CNBD (no./mm^2^)	5^th^ centile CNFL (mm/mm^2^)
Male	16.69 (16.48, 18.75)	16.67 (15.44, 18.75)	12.87 (11.39,13.43)
Female	18.75 (15.62, 19.60)	18.75 (16.40, 21.17)	13.57 (12.72, 14.22)

Data are presented as 0.05^th^ quantile values (90% confidence intervals) for corneal nerve parameters.

## Data Availability

The datasets used to support the findings of this study are available from the corresponding author upon request.

## Supplementary Materials

Supplementary Materials Table 1: neurological examination characteristics of the 257 healthy volunteers.
