# Supplementary material for: Corneal Nerve Parameter Reference Values for Chinese Adults Assessed by Corneal Confocal Microscopy
Source: J Diabetes Res. 2022 Feb 27;2022:4913031. doi: 10.1155/2022/4913031 (PMC8898861; doi:10.1155/2022/4913031)
Supplement: Supplementary Materials — Table 1: neurological examination characteristics of the 257 healthy volunteers. [file 4913031.f1.docx]

**Supplementary material:**

**Table 1** Neurological examination characteristics of the 257 healthy volunteers.

| Neurological examination characteristics | Healthy volunteers(n=257) |
| --- | --- |
| Right SNCV (m⁄ s) | 56.21±4.99  55 (45-66) |
| Right SNAP (μV) | 16.29±5.14  16 (5-32) |
| Left SNCV (m⁄ s) | 56.07±5.26  56 (44-66) |
| Left SNAP (μV) | 16.54±5.35  16 (5-32) |
| vibration sensation (+/-) ^a^ | (0/257) |
| Temperature sensation (+/-) | (0/257) |
| 10-g Semmes-Weinstein monofilament examination (+/-) | (0/257) |
| superficial pain sensation (+/-) | (0/257) |
| ankle jerk reflex (+/-) | (0/257) |
| NSS | 0 |
| NDS | 0 |
| MNSI | 0 |
| TCSS | 0 |

The data are expressed as the means ± SD and median (range). SNCV, sural nerve conduction velocity; SNAP, sural nerve amplitude; NSS, neuropathy symptom score; NDS, neuropathy disability score; MNSI, Michigan neuropathy screening instrument; TCSS, Toronto clinical scoring system. ^a^: Neurological examinations were performed on both legs, any abnormality in one leg is considered positive (+). Normal results on both legs are considered negative (-). Results are expressed as the number of positive/negative (+/-).
